# Patterns of antimicrobial use in Qatar's hospitals: Results from the first national point prevalence survey

**DOI:** 10.1016/j.ijid.2025.108030

**Published:** 2025-10

**Authors:** Jameela Ali Alajmi, Dhouha Hamdani, Eman Khairy Zaky Radwan, Olena Komarcheva, Mohamed Sarhan, Rekayahouda Baaboura, Sheela Shneezai, Syed Hassan Bin Usman Shah

**Affiliations:** 1 Ministry of Public Health (MOPH), Qatar; 2 Hamad Medical Corporation (HMC), Qatar; 3 WHO EMRO Office, Egypt

**Keywords:** Antimicrobial resistance, Antimicrobial utilization, Point prevalence survey, AWaRe classification

## Abstract

**Background and Objectives:**

Antimicrobial resistance (AMR) poses a serious global public health crisis. Optimizing antimicrobial use is critical to slowing the occurrence and spread of resistant pathogens.

**Methods:**

Between September 9 and 27, 2022, a point prevalence survey (PPS) for antimicrobial use was conducted in 17 hospitals in Qatar. All patients admitted to the hospital wards at 8:00 a.m. on survey day constituted the sample. Data included patients’ characteristics, antimicrobials received, and therapeutic indications.

**Results:**

Among the 1733 inpatients surveyed, 811 (46.8%) received at least one antimicrobial. Of 1158 antimicrobial prescriptions, antibacterial agents constituted 82.9% (n = 961), 913 systemic antibacterials and 48 antimycobacterial agents. Parenteral administration predominated (83.3%, n = 676). Indications were documented for 96.3% (n = 1116) of prescriptions. Antimicrobials were prescribed for community-acquired infections (41.4%, n = 479), followed by medical prophylaxis (16.8%, n = 194). Third-generation cephalosporins (16.8%) and penicillin/ β-lactamase inhibitor combinations (15.5%) were most frequently prescribed. For community-acquired infections, penicillin combinations (22%) and third-generation cephalosporins (21%) were preferred. First-generation cephalosporins comprised 46% of surgical prophylaxis prescriptions. WHO AWaRe classification showed 35.5% Access and 40.4% Watch group prescriptions.

**Conclusion:**

Antimicrobial use in Qatar hospitals was generally high, with broad-spectrum antimicrobials predominating. These findings underscore the urgent need for sector-specific targeted antimicrobial stewardship programs to optimize usage.

## Highlights

• First national point prevalence survey on antimicrobial use conducted in Qatar.
• Nearly half (46.8%) of hospitalized patients were receiving at least one antimicrobial.
• Broad-spectrum agents, including 3rd-gen cephalosporins, were commonly prescribed.
• A total of 40.4% of prescriptions belonged to the WHO Watch group, indicating stewardship need.
• Findings provide essential baseline data to inform national antimicrobial policies.

## Introduction

Antimicrobial resistance (AMR) is adversely impacting global health by threatening effective infection treatment and prevention [1]. In 2019, AMR was attributed to 1,270,000 deaths globally [2] and imposes substantial economic burdens on healthcare systems [3]. If unaddressed, AMR could lead to 10 million deaths annually by 2050 and cost US$100 trillion globally [4]. Understanding the trends and magnitude of antimicrobial use in hospitals is crucial, as overuse and misuse drive AMR, with healthcare facilities harboring and disseminating resistant and hard-to-treat pathogens [5]. Increasing reliance on broader-spectrum antibiotics unnecessarily escalates healthcare-associated infections and the AMR crisis [6,7]. Restricting inappropriate antimicrobial use is critical to slowing the spread of resistant pathogens and may curb the development of AMR.

Understanding antimicrobial prescribing patterns and enhancing appropriateness of use are imperative, given the worsening AMR burden in recent decades [8]. However, data on antimicrobial prescribing practices remain limited. The World Health Organization (WHO) has developed the Global Action Plan to ensure effective infection treatment and prevention with quality-assured medicines [9]. The point prevalence survey (PPS) method provides practical surveillance of patient-level antimicrobial use, prescribing patterns, and AMR prevalence [10]. PPS offers valuable snapshot to optimize antimicrobial use, monitor stewardship effectiveness, and strengthen infection control programs, informing policy development and national AMR reduction efforts [11].

Antibiotic prescribing patterns vary regionally. Data on antibiotic use in the Eastern Mediterranean Region (EMRO) is limited, with only seven countries conducting WHO-collaborative PPS [12].

Antimicrobial stewardship (AMS) programs are critical for health system strengthening [13]. The WHO’s AWaRe classification optimizes antibacterial use by categorizing antibiotics into Access [narrow-spectrum), Watch (broad-spectrum), and Reserve (last resort) groups [14], supporting the global target of 60% Access category consumption [15].

Prescribing patterns and AMS efforts in Qatar are not widely documented. Recognizing the need for robust baseline data to guide antimicrobial stewardship interventions, we conducted the first national PPS in collaboration with the WHO-EMRO AMR Unit. The primary objectives were to determine the prevalence of antibiotic use, identify common drug types, evaluate prescribing patterns, and indications among inpatients in public and private hospitals in Qatar. This surveillance aimed to generate evidence to inform national AMS policies and targeted interventions to optimize antimicrobial use.

## Methodology

### Study design and settings

The WHO protocol for PPS [16] was applied for this single-day cross-sectional survey among inpatients at 17 healthcare facilities (government, semi-government, and private) in Doha, Qatar, covering the period between September 9 and 27, 2022. Prior to initiating the survey, infection control personnel from the participating hospitals thoroughly discussed and reached a consensus on the implementation of the protocol. The list of participating hospitals with their bed capacities is provided in Supplementary Table 1.

Sample size was calculated based on expected antimicrobial use prevalence of 50% (±5% precision, 95% confidence interval), requiring 384 patients. Accounting for hospital clustering (intracluster correlation coefficient [ICC]=0.07) and potential non-response (10%), we targeted 1800 patients across 17 hospitals. The final sample of 1733 patients provided adequate power for primary analyses and subgroup comparison.

### Data sources and study population

#### Hospital and ward selection

Hospitals participated voluntarily, based on their interest and capacity to implement the survey, resulting in the inclusion of 17 hospitals. A couple of major tertiary care facilities (representing approximately 20-25% of Qatar’s hospital bed capacity) were excluded due to ongoing infrastructure projects and conflicting research priorities at the time of survey. Representation was ensured from all sectors, comprising ten government hospitals, two semi-government hospitals, and five private hospitals.

All hospital wards (units or departments) within the participating hospitals were included in the survey. Each ward was surveyed only once, on a single day, to calculate the denominator (number of admitted eligible patients with completed forms). However, different wards within the same hospital were surveyed on different days. To minimize day-of-week bias, we ensured that each hospital type (government, semi-government, private) was surveyed across different days of the week, with weekend surveys avoided in surgical wards to ensure adequate elective surgery representation.

A ward assessment tool was used to collect data on ward type, total number of patients, and number of eligible patients (inpatients hospitalized in the ward) at 8:00 a.m. of the ward surveyed on the day of the survey. According to the bed capacity, the hospitals were divided into three categories: less than 200 beds (13 hospitals), 200 to 400 beds (2 hospitals) and more than 400 beds (2 hospitals).

Non-acute wards, such as the long-term care wards (nursing homes or post-treatment centers), accident and emergency departments, day-surgery wards, and day-care wards (e.g., renal dialysis units or endoscopy), were excluded from the survey. Within a single mixed-function ward, patients meeting the above-mentioned inclusion criteria (e.g., in nephrology ward, kidney transplantation patients) and those falling under exclusion criteria (e.g., outpatients undergoing day-care dialysis) could co-exist. A ward was classified as an inpatient ward and included in the survey only if ≥80% of its patients met the inclusion criteria; otherwise, it was excluded. Wards categorization is detailed in the Supplementary Table 2.

Data collection in each hospital was completed within a maximum of three consecutive weeks from the start date. The survey duration varied based on the hospital size and the composition of the survey team. To minimize the impact of unexpected events (*e.g.,* staff holidays) during the survey, it was advised to keep the duration as short as possible. Each ward was completely surveyed within one day to reduce the effect of patients’ movements between wards and within the hospital. The hospital survey team capacity was adequately sized to meet this need.

As the data collection form includes surgical prophylaxis information for the 24-hour period prior to the survey in surgical wards, it was recommended to avoid auditing surgical wards on the day following a weekend or public holiday, as elective procedures may be reduced on these days.

#### Study population

Patients were eligible for inclusion in the survey if they were admitted to a ward by 8:00 a.m. on the day of the survey, irrespective of whether they were on antimicrobials or not. Data collection utilized existing medical records of all admitted eligible patients, capturing clinical and basic epidemiological data and was anonymized using unique identifiers. The numerator comprised all inpatients receiving antimicrobial agents at 8:00 a.m. on the survey day. Patients transferred to another ward after 8:00 a.m. were analysed under their initial ward of admission. Neonates born and admitted before 8:00 a.m. on the day of the survey were included as discrete study subjects. Patients admitted to any of the wards after 8:00 a.m. were excluded.

#### Antimicrobial/antibiotic use

Antimicrobials are classified according to the Anatomical Therapeutic Chemical (ATC) codes developed by the WHO Collaborating Center for Drug Statistics Methodology [17], which categorizes drugs at the chemical group level. Only antimicrobials administered through oral, parenteral, or rectal routes were included in the survey. Topical antimicrobials were excluded. A detailed description of the collection criteria of antibiotic data is given in Supplementary Table 3.

## PPS implementation and data collection

The detailed patient data and hospital- and ward-level data on antimicrobial use were collected using the WHO PPS survey method [16]. Patient data were systematically extracted from medical records through standardized chart reviews by trained infection prevention and control professionals and clinical pharmacists. Clarification of unclear contents was obtained from on-duty medical staff. The multidisciplinary PPS surveillance team comprised infectious disease physicians, clinical microbiologists, clinical pharmacists and hospital infection control nurses/officers.

Data quality was ensured through multiple measures: (1) dual data extraction by clinical pharmacists and infection control nurses for 10% of records with 95% concordance; (2) real-time clarification of unclear entries with bedside nurses; (3) cross-validation of antimicrobial administration with pharmacy dispensing records where available; (4) daily data completeness checks with immediate correction; and (5) final data audit by senior clinical pharmacist before analysis.

To ensure standardization of data collection procedures, a dedicated Android-based application was developed specifically for the PPS. Prior to implementation, all survey team members underwent comprehensive 3-day training. The training included detailed instruction on the study protocol, knowledge of antimicrobials and infections, and the use of the application, delivered through structured workshop sessions with clinical vignettes for consistent application of infection definitions.

The patient-level collected information encompassed demographic details, admission dates, reason for admission, indication for the antimicrobial prescription, and diagnosis. Additionally, a prescribing chart review identified antimicrobial administration during the survey period and subsequently categorized it according to the antimicrobial drug class. The hospital-level data provided general information on the type and size of the hospitals and antimicrobial policies and prescribing practices at hospitals. The ward-level data included information on ward type and the total number of patients admitted on the day of survey.

## Study outcomes

The primary outcome of this study was antimicrobial prescription and utilization during hospitalization. Antimicrobial use was defined as administering or dispensing at least one antimicrobial dose, as documented in hospital records and patient charts. This outcome was evaluated across all patients whose records were reviewed during the survey and corresponds to the sum of the patient case reports completed at the end of the survey period.

The collected data encompassed the clinical rationale for prescribing antimicrobials, including specific diagnoses, indication categories (therapeutic or prophylactic), and type of infection (healthcare-associated or community-acquired). Antimicrobial data includes information on the antimicrobials given to the patient, such as the type of antimicrobials, dose, dosing frequency, route of administration, and other relevant parameters. Additionally, the data provide information on the concordance of the prescribed antimicrobial regimen with established guidelines or procedures applied within the healthcare facilities. Each antimicrobial can be associated with no indication (where no related indication for the given antimicrobial exists), a single indication, or multiple indications (if the same antimicrobial is prescribed to treat multiple co-infections).

The secondary outcome was to assess the categorization of the prescribed antibiotics according to the WHO Access, Watch, Reserve (AWaRe) classification [18]. The AWaRe classifies antibiotics into three stewardship groups: The ``Access'' group contains narrow-spectrum antibacterials, recommended as a first and second choice for most common clinical infection syndromes. The ``Watch'' group contains a broader spectrum of antibacterial classes. The ``Reserve'' group consists of last-resort antibacterial for targeted use in multidrug-resistant infections.

## Statistical analysis

Descriptive statistics were used to report the distribution of the characteristics among admitted patients who met our inclusion criteria. The number and proportion of individual patients with a recorded history of antimicrobial use (defined as receiving at least one antimicrobial agent by one route of administration on the day of the survey) were calculated for all patients admitted by 8:00 a.m. on the day of the survey. The denominator was the number of all patients hospitalized on the day of survey before or at 8:00 a.m. To account for patient clustering within hospitals, we calculated design effects and performed sensitivity analyses using cluster-robust standard errors. The intracluseter correlation coefficient for antimicrobial use was 0.07, indicating a modest but non-negligible clustering effect at the hospital level, supporting the use of cluster-adjusted analyses.

Stratified analyses of antimicrobial use prevalence were conducted across various institutional parameters, including hospital type, institutional affiliation, ward classification, and facility size. Statistical comparison between hospital types were performed using chi-square tests for categorical variables and t-test for continuous variables, adjusted for multiple comparisons using Bonferroni correction. Additionally, logistic regression was used to evaluate the association between hospital affiliation (government, semi-government, private) and the odds of antimicrobial use, with results reported as odds ratio (OR) and 95% confidence intervals (CIs). Government hospitals served as the reference category in the model. A separate binomial logistic regression was conducted using aggregated prescription counts to compare the likelihood of Watch group antibiotic use across hospital types.

Further, prescribed antimicrobials were calculated based on the indications, route of administration, treatment duration, diagnosis site and antibiotic classes. Antimicrobial prescription rates were expressed as a percentage of patients receiving them, or as a percentage of all antimicrobial prescriptions (proportional use). The distribution of prescribed antibacterials by AWaRe classification was calculated to emphasize the significance of appropriate use and monitoring the effect of the stewardship program. Compliance with the treatment guidelines was estimated (manuscript under development). All analyses were performed using STATA software version 17.0 (College Station, TX, USA).

## Results

### Study participants

On the survey days, 1733 inpatients across 17 hospitals were identified eligible and included in this PPS. Overall, the mean age was 35 years (SD ± 24.6), 51.9% (n = 900) were male, 83.2% (n = 1443) were admitted to government hospitals, majority being admitted to the medical ward (36.4%, n = 631), followed by surgical ward and ICU admissions (23.9%, n = 415 and 17.6%, n = 305, respectively). Among all surveyed patients, 94.2% (n = 1634) had documented invasive device insertion (including the peripheral vascular catheter, n = 1134). The median number of days to the survey date from the index admission was three days (interquartile range [IQR] 2-11 days). The demographic characteristics of admitted patients, overall and stratified by antimicrobial use, are summarized in Table 1.

**Table 1:** Demographic characteristics of surveyed patients and the prevalence of antimicrobial use, overall and stratified by antimicrobial use (n = 1733).

Characteristics	All admitted patients at the time of survey	Patients on antimicrobials
	n (%)	n (%)*
Total number of surveyed patients	1733 (100)	811 (46.8)
Mean age of surveyed patients (±SD)	35 ± 24.6	38 ± 23.9
**Gender**		
Male	900 (51.9)	453 (50.3)
Female	833 (48.1)	358 (42.9)
**Admission specialty**		
Medical	631 (36.4)	296 (46.9)
Pediatrics & Neonates	136 (7.8)	38 (27.9)
Surgery	415 (23.9)	254 (61.2)
Obstetrics /maternity	218 (12.6)	69 (31.7)
Critical care (ICU, HDU)	305 (17.6)	137 (44.9)
Gynecology	28 (1.6)	17 (60.7)
Length to stay until day of PPS, days median (IQR)#	3 (2-11)	4 (2-10)
**Antimicrobial use by ward type**		
Adult medical ward	516 (29.7)	210 (40.6)
Adult surgical ward	342 (19.7)	200 (58.4)
Mixed ward	318 (18.3)	135 (42.4)
Neonatal intensive care unit	179 (10.3)	51 (28.4)
Adult high-risk ward	139 (8.0)	81 (58.2)
Adult intensive care unit	107 (6.1)	65 (60.7)
Pediatric medical ward	62 (3.5)	33 (53.2)
Pediatric intensive care unit	39 (2.2)	24 (61.5)
Neonatal medical ward	17 (1.0)	0
Pediatric high-risk ward	14 (1.0)	12 (85.7)
**Use of invasive devices**		
Central vascular catheter	213 (12.3)	151 (70.9)
Peripheral vascular catheter	1134 (65.4)	630 (55.6)
Endotracheal tube	101 (5.8)	68 (67.3)
Urinary catheter	186 (10.7)	126 (67.7)
Hospital affiliation		
Government	1443 (83.2)	647 (44.8)
Semi-Government	169 (9.7)	76 (44.9)
Private	121 (6.9)	88 (72.7)
Antimicrobial use by hospital bed size capacity		
Less than 200	679 (39.1)	362 (53.3)
From 200 to 400	507 (29.2)	167 (32.9)
More than 400	547 (31.5)	282 (51.5)

⁎ Row percentages.

# IQR is interquartile range.

### Antimicrobial use among admitted patients

Out of 1733 inpatients, 811 (46.8%) with a mean age of 38 years (SD ± 23.9) received at least one antimicrobial agent on the day of the survey. The 811 treated patients received 1158 antimicrobials, of which 961 (82.9%) were antibacterial agents, including 913 systemic antibacterials and 48 antimycobacterial agents. A combination of 2 antimicrobials was prescribed in 253 patients, three antimicrobials in 65 and four antimicrobials in 23 patients. Laboratory samples for culture examination were sent for a total of 404 patients (34.8% of those receiving antimicrobials), with culture reports available for 296 of these patients (73.3% of samples sent). Of the total cohort, antimicrobials were prescribed in 453/900 (50.3%) males (Table 1). The median number of days from the index admission to the survey date was 4 days (IQR 2-10). Among patients requiring invasive medical devices, antimicrobial use was observed in 70.9% (151/213) of patients with central line insertions, 67.7% (126/186) with urinary catheter insertions, and 67.3% (68/101) with endotracheal tube insertions.

### Antimicrobial prescriptions by hospital size, affiliation, and ward admissions

Antimicrobial use was notably higher in hospitals with a capacity of less than 200 beds (53.3%, n = 362) and those with a capacity exceeding 400 beds (51.5%, n = 282) compared to hospitals with 200 to 400 beds, where use was comparatively lower (32.9%, n = 167) (Table 1, Supplementary Figure 1). Hospital-specific proportions of patients receiving antimicrobials ranged from 44.8% (647/1443) in government hospitals to 72.7% (n = 88/121) in private hospitals (adjusted odds ratio [aOR = 1.48, 95% CI: 1.14-1.83]) (Table 1, Supplementary Figure 2, Supplementary Tables 4 and 5). However, the cluster-adjusted prevalence of antimicrobial use was 46.8% (95% CI: 32.6-61.5) vs. unadjusted 46.8% (95% CI: 44.5-49.1%). Among all patients admitted across various specialties, antimicrobial prescription rates were highest in the surgical (61.2%, n = 254) and gynecology wards (60.7%, n = 17), followed by patients in the medical ward (46.9%, n = 296) and those admitted to critical care units (16.8%, n = 137). Detailed antimicrobial use according to the hospital and ward type is given in Table 1 and Supplementary Figures 1 and 2.

### Antimicrobial prescription rationale and indications

**Figure 1:**
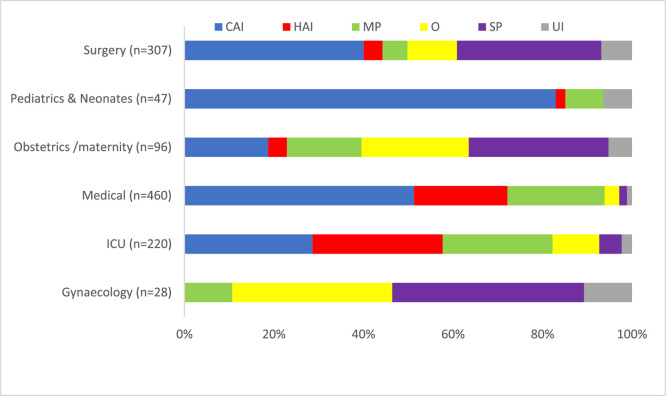
Different indications of prescribed antimicrobials by admission specialty (n = 1158). Abbreviations: CAI, community acquired infections; HAI, healthcare acquired infections; MP, medical prophylaxis; O, other indication; SP, surgical prophylaxis; UI, Unknown indication.

Indications were documented for 96.3% (n = 1116) of the 1158 administered antimicrobial prescriptions, with the treatment of community-acquired infection being the most frequently recorded indication (41.4%, n = 479), followed by medical prophylaxis (16.8%, n = 194). Healthcare-associated infections accounted for 15.4% (n = 178) of the total prescriptions. Among the 160 surgical patients, 20.0% (n = 32) received multiple prophylactic dose prior to surgery. The parenteral route was the predominant mode of administration, utilized in 76.7% (n = 888) of patients, while only 23.1% (n = 267) received oral antimicrobials (Table 2). Detailed antimicrobial indications across different admission specialties are illustrated in Figure 1.

**Table 2:** Indications, route of administration and diagnosis site of antimicrobial prescriptions (n = 1158).

Characteristics	No. of antimicrobials
	n (%)
Total no. of prescribed antimicrobials*	1158 (100)
**Indication**	
Treatment of Community acquired infection	479 (41.4)
Medical Prophylaxis^⁎⁎^	194 (16.8)
Treatment of Healthcare associated infection	178 (15.4)
Surgical prophylaxis	160 (13.8)
Other indication	105 (9.1)
Unknown indication	42 (3.6)
**Route of administration**	
Parenteral	888 (76.7)
Oral	267 (23.1)
Inhalation	3 (0.3)
Rectal	0 (0)
**Diagnosis site (for treatment indication, n = 657)**	
Respiratory infection	197 (30.0)
Gastrointestinal infections & intra-abdominal sepsis	92 (14.0)
Cellulitis, wound, deep soft tissue not involving bone, not related to surgery	79 (12.0)
Clinical sepsis, excluding febrile neutropenia	44 (6.7)
Urinary tract infections	43 (6.5)
Surgical site infections	35 (5.3)
Laboratory-confirmed bacteremia	29 (4.4)
Septic arthritis, osteomyelitis, not related to surgery	24 (3.7)
Febrile neutropenia	22 (3.3)
Obstetric or gynecological infections, STD in women	21 (3.2)
Infections of the central nervous system	19 (2.9)
Systemic inflammatory response with no clear anatomical site	14 (2.1)
Completely undefined; site with no systemic inflammation	9 (1.4)
Infections of ear, nose, throat, larynx and mouth	4 (0.6)
Cardiovascular infections: endocarditis, vascular graft	4 (0.6)
Prostatitis, epididymo-orchitis, STD in men	4 (0.6)
Endophthalmitis	0 (0)
Not applicable	17 (2.6)

*There was one Antimicrobial with unknown name.

^⁎⁎^Medical indications: Indications for medical prophylaxis include prevention of opportunistic infections in immunocompromised patients (e.g., HIV/AIDS patients), prevention of bacterial infections in patients with late-stage cirrhosis, patients with recurrent urinary tract infections, upper gastrointestinal bleeding and acute necrotizing pancreatitis.

Table 2 summarizes the antimicrobial prescription patterns by site of infection. Of the identified diagnoses, respiratory tract infections constituted the primary indication for antimicrobial therapy, accounting for 30% (n = 197) of prescriptions. This was followed by gastrointestinal infections at 14% (n = 92) and skin and soft tissue infections 12% (n = 79). Therapeutic prescriptions for surgical site infections accounted for 5.3% (n = 35) of antimicrobial use (Table 2).

### Antimicrobials selection

**Figure 2:**
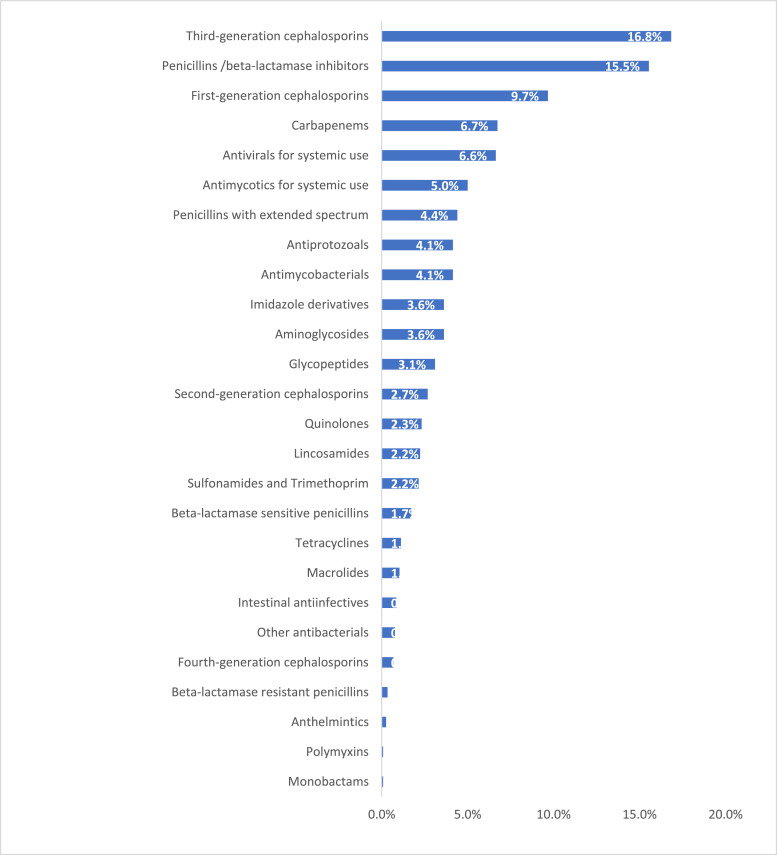
Distribution of antibacterials prescribed during the survey (n = 1158)*. *There is one record with unknown name.

During the PPS, a total of 1158 antimicrobial prescriptions were documented; of these, antibacterials for systemic use accounted for 78.8% (n = 913) prescriptions. Considering exclusively the prescribed antibacterials, third-generation cephalosporins emerged as the most frequently prescribed class, comprising 16.8% (195/1158) of prescriptions. This was followed by penicillin/ β-lactamase inhibitors combinations (15.5%, 180/1158), first-generation cephalosporins (9.7%, 112/1158) and carbapenems (6.7%, 78/1158) (Figure 2). For community-acquired infections, combinations of penicillins-/β-lactamase inhibitors (22%) and third-generation cephalosporins (21%) were the preferred treatments. A comprehensive description of antibacterial and other antimicrobial prescriptions by indication is provided in Supplementary Table 6. For surgical prophylaxis, first-generation cephalosporins accounted for 46% (74/160) of all prescriptions, reflecting their predominant use in this context. The prescription rate of third-generation cephalosporins was particularly high in private hospitals, reaching 66% (58/88) (Supplementary Table 6).

*Among nonantibacterial antimicrobials, antivirals were the most prescribed drugs, accounting for 6.6% (77/1158) of the total prescribed antimicrobials. This was followed by antifungal agents, which represented 5% (58/1158), and antiprotozoals accounted for 4.1% (48/1158) of all prescriptions. In contrast, the prescription rates for anthelmintic and antimalarial agents were notably low (Supplementary Table 6).*

### Prescribed antimicrobials by AWaRe classification

**Figure 3:**
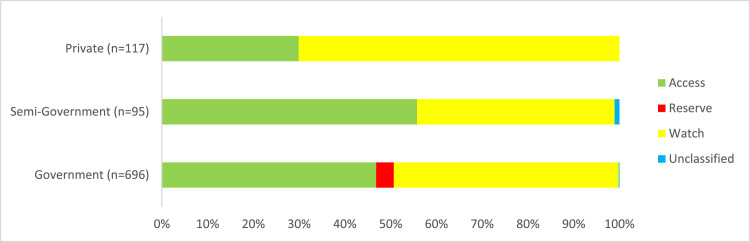
Percentage of antibiotic use according to the WHO AWaRe classification by hospital affiliation (n = 908).

Figure 3 illustrates the patterns of antibiotic use according to the WHO AWaRe classification. Of the 1158 antimicrobials prescribed, 38.0% (n = 441), 40.4% (n = 468), and 2.3% (n = 27) fell respectively under the Access, Watch, and Reserve group.

The prescribing patterns of antibiotics from the Watch group differed markedly between the public and private hospitals. Compared to the public sector (48.8%, 340/696), the private sector hospitals had a significantly higher proportion of the Watch group antibiotics use (70.0%, 82/117) (aOR = 2.51, 95% CI: 1.92-3.09) (Supplementary Table 7). Reserve group antibiotics were only used in the public sector hospitals (Figure 3).

## Discussion

The global burden of AMR continues to escalate, primarily driven by the inappropriate and overuse of antimicrobials [6]. This first comprehensive large-scale PPS in Qatar demonstrates a high antimicrobial consumption rate (46.8%), despite the government-implemented stewardship programs since 2019. The overall antimicrobial consumption rate was higher in the private sector (72.7%) compared to the governmental (44.8%) and semi-governmental (44.9%) healthcare setups. Encouragingly, 35.4% and 40.4% of prescriptions were from WHO AWaRe Access and Watch groups, respectively. Private hospitals showed significantly higher Watch group antibiotics use (70%). These findings, particularly those outlining facilities with higher antimicrobial prescription patterns, provide critical insights for public health policies and enhance stewardship efforts.

The marked difference in antimicrobial prescribing between private (72.7%) and government (44.8%) hospitals represents a critical finding requiring targeted intervention. This disparity likely reflects multiple factors including: differential regulatory oversight with private hospitals having less stringent antimicrobial stewardship monitoring; patient population differences with private hospitals serving higher proportion of expatriate population who may have different healthcare expectations; varying physician training backgrounds and prescribing cultures; and potential financial incentives favoring broader-spectrum therapies in fee-for-service models. The significantly higher Watch group antibiotic use in private hospitals (70% vs 48.8%) further emphasizes the urgent need for enhanced stewardship programs and regulatory frameworks in private healthcare settings.

The observed prevalence of antimicrobial use in Qatar (46.8%) is lower than Eastern Mediterranean countries (56.9% across 7 countries and 139 hospitals) [12], Latin America (49.5%) [19], and Africa (>70%) [[20], [21], [22]]; indicating progress in physicians’ awareness regarding the importance of antimicrobial stewardship in clinical practice. However, rates remain higher than that reported in some European countries (32% across 32 countries and 215 hospitals), the Americas (38% across 6 countries and 43 hospitals), and other Asian countries (39% across 15 countries and 56 hospitals) [23], highlighting implementation gaps. The notably higher consumption in private hospitals, particularly Watch group antibiotics, necessitates targeted interventions to strengthen stewardship in these settings, aligning with Qatar’s Third National Health Strategy priorities [24].

The high prevalence of antimicrobial use in Qatar reflects the complex healthcare environment typical of Gulf Cooperation council countries, where rapid healthcare system expansion, diverse expatriate physician workforce, and varying levels of stewardship program maturity create challenges for optimal prescribing. The post COVID-19 context during our survey period may have influenced empirical prescribing patterns, as healthcare systems transitioned from pandemic protocols to standard care practices. However, our findings establish crucial baseline data for longitudinal monitoring and policy development.

Injudicious prescribing prevails in nations with inadequate regulatory frameworks governing antimicrobial utilization and suboptimal stewardship programs [25]. These prescribing patterns are influenced by multiple factors, including the high epidemiological burden of infectious diseases within these jurisdictions and sociocultural determinants that drive heightened patient expectations for antimicrobial therapy [12]. In low-and middle-income countries, the situation is further exacerbated by insufficient physician training on antimicrobial stewardship principles and limited access to microbiological testing within the healthcare facilities. These constraints often compel healthcare providers to rely on empiric treatment strategies, necessitating broad-spectrum antibiotic prescriptions to cover a wide range of potential bacterial pathogens [26]. The interplay between these variables creates a complex healthcare environment that may perpetuate inappropriate antimicrobial use [12]. Given that national- and facility-level infection prevention and control programs are still evolving in Qatar- although IPC is relatively well established within the government sector, it remains underdeveloped in many private healthcare settings- developing locally appropriate treatment guidelines and implementing targeted, institution-based antimicrobial stewardship programs are critical. These initiatives coordinated under the Ministry of Public Health, should be prioritized across healthcare facilities to optimize prescribing practices and mitigate AMR.

Our finding that only 34.8% of patients receiving antimicrobials had microbiological samples sent for culture, with results available for just 73.3% of those samples, highlight a critical diagnostic stewardship gap. This limitation severely constrains clinicians’ ability to practice targeted antimicrobial therapy and likely contributes to the observed predominance of broad-spectrum prescribing. Enhancing diagnostic capabilities, implementing rapid diagnostic technologies, and establishing protocols for systematic culture acquisition should be prioritized alongside traditional antimicrobial stewardship interventions.

Overprescribing antimicrobials will continue generating resistant strains, burdening heavily on the healthcare cost [15]. Enhancing microbiological diagnostics-encompassing improved diagnostic tools accessibility, increased laboratory sample submission, and results-guided treatment optimization-remains crucial. However, laboratory confirmation of culture reports was limited within our sample. Understanding the factors behind progressively increasing antimicrobial prescription upsurge can be vital. Treatment of infections and surgical prophylaxis emerged as primary prescription indications, aligning with previous studies [25,27], with respiratory, gastrointestinal, and skin/soft tissue infections most frequently treated. Given the well-established correlation between the overuse of antimicrobials and subsequent resistance development [13], there is an urgent need to prioritize regular educational workshops for healthcare prescribers and pharmacists, along with health education sessions for the general public focusing on appropriate antimicrobial use and compliance.

Expanding antimicrobial stewardship program coverage is essential for mitigating infectious disease burden. Literature demonstrates these programs effectively optimize antimicrobial use and improve clinical outcomes [14,15]. Adopting the WHO’s AWaRe classification guidelines can serve as a key tool in preventing progressively increasing resistance rates and avoiding therapeutic impasses. Our Access group prescriptions (35.4%) align with Eastern Mediterranean patterns (29% Jordan to 46% Tunisia) [12]. However, this figure remains substantially lower than European countries and the United States (∼68%) [28]. Innovative and context-specific strategies tailored to local healthcare settings are required to achieve the WHO target of 60% prescription from the Access group.

Achieving the WHO target of 60% Access group prescriptions will require systematic interventions addressing both clinical decision-making and healthcare system factors. Specific strategies should include: development of local antimicrobial guidelines adapted to Qatar’s epidemiological context; implementation of electronic prescribing systems with clinical decision support; establishment of pharmacy-led stewardship interventions; mandatory stewardship training for all prescribing physicians; and creation of performance metrics linking stewardship compliance to institutional accreditation and physician credentialing processes.

Third-generation cephalosporins and β-lactam compounds emerged as the most frequently used antibiotics in our survey, aligning with trends reported in other countries [23,29]. High use of broad-spectrum cephalosporins- first and -third-generation, categorized within the Watch group of the AWaRe classification- for surgical and medical prophylaxis raises concerns, contravening established WHO stewardship guidelines [18]. Encouragingly, the proportion of prescriptions from the Reserve group was relatively limited (∼2%) compared to rates reported in Pakistan (∼3%) [12] and Latin America (4.7%) [30], though still exceeding the reported use in high-income countries (1.5%) [28]. Given that Reserve group antimicrobials are typically designated for managing recalcitrant infections, their use warrants careful monitoring to preserve efficacy [15,18]. Additionally, the concurrent use of three or more antimicrobials in a single patient raises significant concerns regarding appropriate prescribing practices. Strengthening antimicrobial stewardship programs should be prioritized and viewed within the framework of the WHO’s AWaRe classification to optimize antimicrobial use and mitigate resistance development [18].

Antibiotic utilization was significantly higher among patients undergoing catheterization, tube insertion, or surgical procedures compared to those without such interventions. This observation may reflect the presence of more severe underlying conditions in these patients or indicate that these procedures could serve as potential sources of nosocomial infections [29]. Additionally, promoting timely parenteral to oral antimicrobial transitions, where clinically appropriate, is crucial. This practice aligns with antimicrobial stewardship principles while minimizing prolonged parenteral therapy risks, including catheter-associated infections and increased healthcare costs [15].

## Policy and implementation implications

The findings from this first national PPS provide a foundation for developing comprehensive antimicrobial stewardship policies in Qatar. Immediate policy priorities should include: (1) mandatory implementation of antimicrobial stewardship programs in all private healthcare facilities with standardized core elements including antimicrobial guidelines, approved processes, and outcome monitoring; (2) enhanced compliance with established national antimicrobial prescribing guidelines tailored to local epidemiology and resistance patterns; (3) integration of stewardship metrics into healthcare facility licensing and accreditation requirements; (4) Strengthening the national antimicrobial surveillance system to enable continuous monitoring and benchmarking across facilities; and (5) adoption of parenteral-to-oral conversion protocols, where clinically appropriate, to reduce unnecessary intravenous therapy and its associated risks.

Given the substantial disparities between sectors, regulatory harmonization is essential, with private hospitals requiring enhanced oversight including mandatory data reporting, standardized stewardship requirements, and financial incentives aligned with appropriate prescribing practices. Government hospitals, while demonstrating better stewardship performance, require continued investment in diagnostic capabilities and physician education to achieve WHO targets. Semi-government facilities should serve as pilot sites for innovative stewardship interventions given their immediate performance metrics.

## Limitations

There are several limitations to our study. First, we assessed antimicrobial use through medical record reviews rather than direct administration confirmation, potentially overestimating prevalence if prescribed antimicrobials were not administered. Medical charts may also contain incomplete information, especially for same-day admissions. Second, is the voluntary participation of hospitals, which may have introduced selection bias. Hospitals with established antimicrobial stewardship programs, better resources, and motivated infection control teams were more likely to participate. This may have resulted in underestimation of true antimicrobial use prevalence across Qatar’s healthcare system. Third, despite investigating 17 hospitals in Qatar, some large tertiary care facilities were excluded. The exclusion of these major tertiary centers (representing approximately 25% of Qatar’s hospital bed capacity) likely resulted in underestimation of broad-spectrum antimicrobial use. Fourth, the single time-point nature of our survey may not capture temporal variations in antimicrobial prescribing, as prescribing patterns may vary by day of admission (weekend vs weekday admission), seasonal factors (respiratory infection seasons), and hospital operational cycles (post-holiday periods, academic year transitions). Moreover, our reliance on medical record documentation from 2022 may reflect post-COVID-19 pandemic prescribing patterns, potentially influencing generalizability to current practice patterns. Fifth, the study design limited direct engagement with physicians and patients, constraining our exploration of underlying factors. Lastly, although the data reflect practices from 2022, as Qatar’s first PPS, these findings remain valuable for establishing benchmarks and monitoring compliance patterns. This survey provides crucial baseline metrics for longitudinal evaluation of stewardship programs. Information on national guidelines-based antimicrobial adherence and use will be available in future.

## Conclusion

Antimicrobial utilization in hospitals across Qatar was notably high, with a predominant reliance on broad-spectrum agents in clinical practice. The majority of Watch group prescriptions demonstrate low compliance with several quality indicators of antimicrobial stewardship. These findings highlight critical needs for targeted, innovative strategies to strengthen stewardship programs, optimize use, and preserve Reserve group antimicrobials. The observed heterogeneity in prescribing patterns necessitates hospital-based internal auditing and a comprehensive national surveillance system to ensure guideline-concordant antimicrobial utilization. Additionally, physicians’ education and training on appropriate prescribing practices are crucial.

The substantial disparities between private and government hospital prescribing patterns underscore the urgent need for sector-specific interventions and regulatory harmonization. Achieving WHO AWaRe targets will require coordinated efforts encompassing enhanced diagnostic stewardship, mandatory implementation of prescribing stewardship programs across all healthcare sectors, and systematic physician education initiatives. These baseline findings establish Qatar's position for regional leadership in antimicrobial stewardship while highlighting the critical importance of sustained policy commitment and resource allocation for long-term AMR mitigation.

## Funding

This research did not receive any specific grant from funding agencies in the public, commercial, or not-for-profit sectors.

## Ethical approval

The Ministry of Public Health, Qatar (ERC-823-3-2022) and WHO EMRO region office and hospital authorities provided permission to conduct this PPS. Given that this PPS was conducted as a public health surveillance activity and involved solely a retrospective review of medical records without direct patient involvement, the institutional review boards of the participating healthcare facilities granted a waiver of informed consent. Approval was obtained from the respective facility leadership.

## Author contributions

JAA, DH, EKZR, OK, MS and RB contributed to study conception and design, data acquisition and analysis, interpretation of findings, and drafting of the manuscript; JAA, MS, SS, and SHBUS contributed to data acquisition and analysis and interpretation of findings. JAA, MS wrote the first draft and SHBUS revised it. All the authors revised the manuscript for final approval.

## Declaration of competing interest

The authors declare that they have no known competing financial interests or personal relationships that could have appeared to influence the work reported in this paper.

## Appendix Supplementary materials

Image, application 1

Image, application 2
