# Supplementary material for: Patterns of antimicrobial use in Qatar's hospitals: Results from the first national point prevalence survey
Source: Int J Infect Dis. 2025 Oct;159:None. doi: 10.1016/j.ijid.2025.108030 (PMC12485078; doi:10.1016/j.ijid.2025.108030)
Supplement: Supplementary file 1 [file mmc1.docx]

Supplementary Tables and Figures

**Supplementary Table 1. List of selected hospitals with number of beds and affiliation**

| S/no | **Name** | **# of Beds** | **Specialty** | **Location** | **Affiliation** |
| --- | --- | --- | --- | --- | --- |
| 1 | Communicable Disease Center (CDC) | 50 | Infectious Diseases | Doha | Governmental Hospitals |
| 2 | National Center for Cancer Care and Research (NCCCR) | 61 | Cancer | Doha | Governmental Hospitals |
| 3 | Cuban Hospital | 72 | General | Zekreet | Governmental Hospitals |
| 4 | Heart Hospital (HH) | 114 | Cardiac | Doha | Governmental Hospitals |
| 5 | Al-Khor Hospital (AKH) | 115 | General | Al-Khor | Governmental Hospitals |
| 6 | Rumailah Hospital (RH) | 222 | Plastic Surgery | Doha | Governmental Hospitals |
| 7 | Al-Wakrah Hospital (AWH) | 288 | General and Burn Unit | Al-Wakrah | Governmental Hospitals |
| 8 | Women’s Wellness and Research Center (WWRC) | 339 | Women/Obstetrics and Gynecology | Doha | Governmental Hospitals |
| 9 | Hamad General Hospital (HGH) | 950 | General | Doha | Governmental Hospitals |
| 10 | Hazm Mebaireek General Hospital | 103 | General | Al-Rayyan | Governmental Hospitals |
| 11 | Aspetar | 22 | Orthopedic and Sports Medicine | Doha | Semi-Governmental Hospital |
| 12 | Sidra Medicine and Research Center | 281 | Obs/ Gyn & Tertiary Care Pediatric | Doha | Semi-Governmental Hospital |
| 13 | Aster | 33 | General | Doha | Private hospitals |
| 14 | Al-Emadi Hospital (AEH) | 47 | General | Doha | Private hospitals |
| 15 | Doha Clinic Hospital (DCH) | 54 | General | Doha | Private hospitals |
| 16 | Turkish Hospital | 65 | General | Doha | Private hospitals |
| 17 | Al-Ahli Hospital (AAH) | 134 | General | Doha | Private hospitals |

**Supplementary Table 2. Classification of wards based on specialty and care requirements**

| **Category** | **Ward types** |
| --- | --- |
| Pediatric departments | - Pediatric Medical Ward  - Pediatric Surgical Ward  - Pediatric High-Risk Ward  - Pediatric Intensive Care Unit |
| Neonatal departments | - Neonatal Medical Ward  - Neonatal Intensive Care Unit |
| Adult departments | - Adult Medical Ward  - Adult Surgical Ward  - Adult High-Risk Ward  - Adult Intensive Care Unit |
| Mixed departments/ wards | - Wards where patients belong to different specialty categories within the same ward |
| High-Risk Wards | - Wards with high antibiotic consumption due to the nature of care, such as:  - Hematology  - Oncology  - Burns  - Transplantation  - Infectious Diseases |

**Supplementary Table 3. Collection criteria of data on antimicrobial use and dosage**

| **Criteria** | **Sub-category** | **Details** |
| --- | --- | --- |
| Inclusion criteria for antimicrobials therapy (treatment or prophylaxis) | Timing requirements | Include if the patient is under ongoing antimicrobials therapy at 8.00 a.m. on the day of the survey  Exclude if the antibiotic therapy started after 8.00 a.m. on the day of the survey  Exclude if the antimicrobials therapy was stopped before 8.00 a.m. on the day of the survey |
|  | Included antimicrobial agents | 1. Antimicrobials for systemic use 2. Antimycotics and antifungals for systemic use 3. Drugs for treatment of TB 4. Antibiotics used as intestinal anti-infectives 5. Antiprotozoal used as antibacterial agents, nitroimidazole derivatives 6. Antivirals used for systemic use 7. Antimalarials |
| Special cases of antibiotic therapy | Modified dosing frequency or long-acting antibiotics | Report if the patient is under ongoing treatment due to modified dosing frequency (renal impairment) or with a long-acting antibiotic at 8 a.m., even if the antibiotic is not administered on the day of the survey (e.g., administered every two days). |
|  | Treatment changes | Report antibiotic A if the patient is on antibiotic A at 8 a.m. but changes to antibiotic B at 10 a.m. |
| Collection of data on antibiotics | Naming convention | Antibiotics are reported using the international nonproprietary names (INN) of the substance |
| Dosing | Prescribed daily dose | The prescribed daily dose is an essential parameter for assessing antimicrobials use. This includes information on the unit dose and the frequency of doses administered. |
| Surgical prophylaxis (SP) | Duration categories | 1. SP1: One dose administered 2. SP2: Multiple doses within 24 hours 3. SP3: Multiple doses in more than 24 hours |
|  | Timing | SP1: Surgical prophylaxis was administered within 24 hours from 8 a.m.  SP2: Surgical prophylaxis was administered multiple times within 24 hours from 8 a.m.  SP3: Surgical prophylaxis was administered more than 24 hours before 8 a.m. |

**Supplementary Table 4. Bivariate Analysis of Factors Associated with Antimicrobial Use Using Chi-Square Tests**

| **Characteristics** | **Antimicrobial use** | | **P value** |
| --- | --- | --- | --- |
|  | **Yes n (%)** | **No n (%)** |  |
| **Gender** |  |  |  |
| Male | 453 (55.9) | 447 (48.4) | 0.002 |
| Female | 358 (44.1) | 475 (51.5) |  |
| **Age groups** |  |  |  |
| <18 years | 163 (20.1) | 281 (30.4) | <0.001 |
| 18 – 59 years | 472 (58.2) | 508 (55.1) |  |
| 60+ years | 176 (21.7) | 133 (14.4) |  |
| **Admission specialty** |  |  |  |
| Medical | 296 (36.5) | 335 (36.3) | <0.001 |
| Pediatrics & Neonates | 38 (4.7) | 98 (10.6) |  |
| Surgery | 254 (31.3) | 161 (17.5) |  |
| Obstetrics /maternity | 69 (8.5) | 149 (16.1) |  |
| Critical care (ICU, HDU) | 137 (16.9) | 168 (18.2) |  |
| Gynecology | 17 (2.1) | 11 (1.1) |  |
| **Antimicrobial use by ward type** |  |  |  |
| Adult medical ward | 210 (25.8) | 306 (33.1) | <0.001 |
| Adult surgical ward | 200 (24.6) | 142 (15.4) |  |
| Mixed ward | 135 (16.6) | 183 (19.8) |  |
| Neonatal intensive care unit | 51 (6.3) | 128 (13.8) |  |
| Adult high-risk ward | 81 (10.0) | 58 (6.3) |  |
| Adult intensive care unit | 65 (8.0) | 42 (4.5) |  |
| Pediatric medical ward | 33 (4.0) | 29 (3.2) |  |
| Pediatric intensive care unit | 24 (2.9) | 15 (1.6) |  |
| Neonatal medical ward | 0 (0) | 17 (1.8) |  |
| Pediatric high-risk ward | 12 (1.5) | 2 (0.2) |  |
| **Use of invasive devices** |  |  |  |
| Central vascular catheter | 151 (18.6) | 62 (6.7) | <0.001 |
| Peripheral vascular catheter | 630 (77.6) | 504 (54.6) |  |
| Endotracheal tube | 68 (8.3) | 33 (3.5) |  |
| Urinary catheter | 126 (15.5) | 60 (6.5) |  |
| **Hospital affiliation** |  |  |  |
| Private | 88 (10.8) | 33 (3.5) | <0.001 |
| Semi-Government | 76 (9.3) | 93 (10.9) |  |
| Government | 647 (79.7) | 796 (86.3) |  |
| **Antimicrobial use by hospital bed size capacity** |  |  |  |
| More than 400 | 282 (34.7) | 265 (28.7) | <0.001 |
| From 200 to 400 | 167 (20.5) | 340 (36.8) |  |
| Less than 200 | 362 (44.6) | 317 (34.3) |  |
| **History of COVID-19** |  |  |  |
| Yes | 35 (4.3) | 25 (2.7) | 0.066 |
| No | 767 (95.6) | 890 (97.2) |  |

**Supplementary Table 5. Multivariable Logistic Regression Analysis of Predictors of Antimicrobial Use**

| **Characteristics** | **Antimicrobial use** | | **P value** |
| --- | --- | --- | --- |
|  | **Adjusted OR** | **95% CI** |  |
| **Gender** |  |  |  |
| Male | - 0.06 | -0.21, 0.08 | 0.368 |
| Female | Ref |  |  |
| **Age in years** | 0.00 | -0.00, 0.00 | 0.285 |
| **Admission specialty** |  |  |  |
| Medical | -0.95 | -1.68, -0.21 | 0.011 |
| Pediatrics & Neonates | -1.37 | -2.26, -0.48 | 0.003 |
| Surgery | -0.34 | -1.46, 0.77 | 0.545 |
| Obstetrics /maternity | -1.04 | -1.68, -0.41 | 0.001 |
| Critical care (ICU, HDU) | -0.07 | -2.54, 2.40 | 0.954 |
| Gynecology | Ref |  |  |
| **Antimicrobial use by ward type** |  |  |  |
| Adult medical ward | -0.78 | -2.72, 1.14 | 0.425 |
| Adult surgical ward | -0.47 | -2.16, 1.22 | 0.587 |
| Mixed ward | -0.96 | -2.33, 0.41 | 0.170 |
| Neonatal intensive care unit | -1.77 | -3.32, -0.23 | 0.024 |
| Adult high-risk ward | Ref |  |  |
| Adult intensive care unit | -1.84 | -3.91, 0.22 | 0.080 |
| Pediatric medical ward | 0.46 | -1.44, 2.38 | 0.633 |
| Pediatric intensive care unit | -1.06 | -3.24, 1.11 | 0.339 |
| Neonatal medical ward | 0 |  |  |
| Pediatric high-risk ward | 1.21 | -0.71, 3.14 | 0.218 |
| **Use of invasive devices** |  |  |  |
| Central vascular catheter (Yes) | 1.45 | 0.86, 2.05 | <0.001 |
| No | Ref |  |  |
| Peripheral vascular catheter (Yes) | 1.10 | 0.08, 2.13 | 0.035 |
| No | Ref |  |  |
| Endotracheal tube (Yes) | 0.23 | -0.07, 0.54 | 0.138 |
| No | Ref |  |  |
| Urinary catheter (Yes) | 0.73 | 0.26, 1.20 | 0.002 |
| No | Ref |  |  |
| **Hospital affiliation** |  |  |  |
| Private | 1.48 | 1.14, 1.83 | <0.001 |
| Semi-Government | -0.57 | -0.86, -0.27 | <0.001 |
| Government | Ref |  |  |
| **Antimicrobial use by hospital bed size capacity** |  |  |  |
| More than 400 | -0.41 | -0.85, 0.02 | 0.067 |
| From 200 to 400 | -.031 | -0.84, 0.21 | 0.248 |
| Less than 200 | Ref |  |  |
| **History of COVID-19** |  |  |  |
| Yes | 0.38 | -0.38, 1.15 | 0.328 |
| No | Ref |  |  |

**Supplementary Table 6. Distribution of prescribed antimicrobials by indications (n=1,158)**

| **Antimicrobial type** | **Community acquired infections** | **Healthcare acquired infections** | **Medical prophylaxis** | **Surgical prophylaxis** | **Other indication** | | **Unknown indication** |
| --- | --- | --- | --- | --- | --- | --- | --- |
|  | n (%) | n (%) | n (%) | n (%) | n (%) | n (%) | |
| **Antibacterial for systemic use (J01)** | **377 (79)** | **161 (90)** | **92 (47)** | **150 (94)** | **92 (88)** | **41 (98)** | |
| First-generation cephalosporins (J01DB) | 15 (3) | 0 | 8 (4) | 74 (46) | 8 (8) | 7 (17) | |
| Second-generation cephalosporins (J01DC) | 7 (1) | 1 (1) | 2 (1) | 17 (11) | 3 (3) | 1 (2) | |
| Third generation cephalosporins (J01DD) | 99 (21) | 9 (5) | 9 (5) | 36 (23) | 27 (26) | 15 (36) | |
| Fourth generation cephalosporins (J01DE) | 2 (0) | 6 (3) | 0 | 0 | 0 | 0 | |
| Aminoglycosides (J01G) | 8 (2) | 7 (4) | 19 (10) | 0 | 8 (8) | 0 | |
| Carbapenems (J01DH) | 29 (6) | 43 (24) | 0 | 1 (1) | 4 (4) | 1 (2) | |
| Quinolones (J01M) | 12 (3) | 8 (4) | 3 (2) | 2 (1) | 2 (2) | 0 | |
| Glycopeptides (J01XA) | 10 (2) | 16 (9) | 4 (2) | 1 (1) | 4 (4) | 1 (2) | |
| Imidazole derivatives (J01XD) | 14 (3) | 2 (1) | 3 (2) | 4 (3) | 13 (12) | 6 (14) | |
| Other Antibacterials (J01XX) | 6 (1) | 2 (1) | 1 (1) | 0 | 0 | 0 | |
| Lincosamides (J01FF) | 20 (4) | 3 (2) | 0 | 2 (1) | 1 (1) | 0 | |
| Macrolides (J01FA) | 9 (2) | 0 | 2 (1) | 0 | 1 (1) | 0 | |
| Penicillins with extended spectrum (J01CA) | 14 (3) | 5 (3) | 19 (10) | 4 (3) | 3 (3) | 6 (14) | |
| Beta-lactamase sensitive Penicillins (J01CE) | 8 (2) | 0 | 7 (4) | 0 | 5 (5) | 0 | |
| Beta-lactamase resistant Penicillins (J01CF) | 3 (1) | 0 | 0 | 0 | 1 (1) | 0 | |
| Penicillins /beta-lactamase inhibitors (J01CR) | 107 (22) | 43 (24) | 5 (3) | 9 (6) | 12 (11) | 4 (10) | |
| Polymyxins (J01XB) | 0 | 1 (1) | 0 | 0 |  | 0 | |
| Tetracyclines (J01AA) | 8 (2) | 5 (3) | 0 | 0 | 0 | 0 | |
| Sulfonamides and Trimethoprim (J01EE) | 5 (1) | 10 (6) | 10 (5) | 0 | 0 | 0 | |
| Monobactams (J01DF) | 1 (0) | 0 | 0 | 0 | 0 | 0 | |
| **Antimycotics for systemic use (J02)** | 4 (1) | 15 (8) | 35 (18) | 0 | 4 (4) | 0 | |
| **Antimycobacterial (J04)** | 45 (9) | 1 (1) | 2 (1) | 0 | 0 | 0 | |
| **Antivirals for systemic use (J05)** | 30 (6) | 1 (1) | 44 (23) | 0 | 2 (2) | 0 | |
| **Antiprotozoals (P01)** | 19 (4) | 0 | 13 (7) | 10 (6) | 5 (5) | 1 (2) | |
| **Anthelmintics (P02)** | 3 (1) | 0 | 0 | 0 | 0 | 0 | |
| **Intestinal Anti-infective (A07A)** | 0 | 0 | 8 (4) | 0 | 2 (2) | 0 | |
| **Unknown** | 1 (0) | 0 | 0 | 0 | 0 | 0 | |
| **Total** | **479** | **178** | **194** | **160** | **105** | **42** | |

**Supplementary Table 7. Multivariable Logistic Regression Analysis of Predictors of Watch Group Use**

| **Characteristics** | **Watch group Antibiotic use** | | **P value** |
| --- | --- | --- | --- |
|  | **Adjusted OR** | **95% CI** |  |
| **Gender** |  |  |  |
| Male | - 0.01 | -0.26, 0.22 | 0.898 |
| Female | Ref |  |  |
| **Age in years** | 0.00 | 0.00, 0.01 | 0.001 |
| **Admission specialty** |  |  |  |
| Medical | 0.59 | -0.19, 1.38 | 0.139 |
| Pediatrics & Neonates | 1.00 | -0.06, 2.07 | 0.067 |
| Surgery | 0.46 | -0.74, 1.68 | 0.448 |
| Obstetrics /maternity | 0.14 | -1.42, 1.71 | 0.860 |
| Critical care (ICU, HDU) | 2.17 | 0.04, 4.29 | 0.045 |
| Gynecology | Ref |  |  |
| **Antimicrobial use by ward type** |  |  |  |
| Adult medical ward | -0.29 | -1.50, 0.92 | 0.639 |
| Adult surgical ward | -0.12 | -1.12, 0.86 | 0.800 |
| Mixed ward | -0.96 | -2.32, 0.40 | 0.169 |
| Neonatal intensive care unit | -3.24 | -4.06, -2.42 | <0.001 |
| Adult high-risk ward | Ref |  |  |
| Adult intensive care unit | -2.13 | -3.53, -0.73 | 0.003 |
| Pediatric medical ward | 0.65 | -0.71, 2.02 | 0.349 |
| Pediatric intensive care unit | -1.26 | -2.10, -0.42 | 0.003 |
| Neonatal medical ward | 0 |  |  |
| Pediatric high-risk ward | 0.56 | -0.66, 1.79 | 0.366 |
| **Use of invasive devices** |  |  |  |
| Central vascular catheter (Yes) | 1.11 | 0.72, 1.50 | <0.001 |
| No | Ref |  |  |
| Peripheral vascular catheter (Yes) | 0.79 | 0.19, 1.39 | 0.010 |
| No | Ref |  |  |
| Endotracheal tube (Yes) | 0.57 | -0.03, 1.17 | 0.063 |
| No | Ref |  |  |
| Urinary catheter (Yes) | 0.34 | 0.19, 0.49 | <0.001 |
| No | Ref |  |  |
| **Hospital affiliation** |  |  |  |
| Private | 2.51 | 1.92, 3.09 | <0.001 |
| Semi-Government | -0.01 | -0.50, 0.47 | 0.948 |
| Government | Ref |  |  |
| **Antimicrobial use by hospital bed size capacity** |  |  |  |
| More than 400 | 0.03 | -0.18, 0.25 | 0.657 |
| From 200 to 400 | 0.06 | -0.23, 0.37 | 0.657 |
| Less than 200 | Ref |  |  |
| **History of COVID-19** |  |  |  |
| Yes | 0.02 | -0.57, 0.63 | 0.933 |
| No | Ref |  |  |

**Supplementary Figures**

**Supplementary Figure 1**

Abbreviations: CDC, Communicable Disease Center; HMGH, Hazm Mebaireek General Hospital; NCCCR, National Center for Cancer Care and Research, WWRC, Women’s Wellness and Research Center

**Supplementary Figure 1. Prevalence of antimicrobial use by hospitals (n=1,733)**

**Supplementary Figure 2**

**Supplementary Figure 2. Prevalence of antimicrobial use by hospital affiliation, overall and by ward admissions (n=1,733)**
