# Supplementary material for: Patterns of antimicrobial use in Qatar's hospitals: Results from the first national point prevalence survey
Source: Int J Infect Dis. 2025 Oct;159:None. doi: 10.1016/j.ijid.2025.108030 (PMC12485078; doi:10.1016/j.ijid.2025.108030)
Supplement: Supplementary file 2 [file mmc2.pdf]

Date: August 23, 2022

Ref.: ERC-823-3-2022

Exempt Research Certificate

Dear Applicant,

The Health Research Governance Department at the Ministry of Public health (MoPH) has reviewed the research project entitled *"Prevalence of Antimicrobial Use in the State of Qatar"*. The Principal Investigator, Eman Radwan, confirmed that there will be no collection of identifiable information. Upon review, the research has been categorized as **exempt research under category (3):** Research involving the collection or study of existing data, documents, records, pathological specimens, or diagnostic specimens, if these sources are publicly available or if the information is recorded by the investigator in such a manner that subjects cannot be identified.

However, please note that in accordance with MoPH policy, the regulations state that "research involving…interview procedures…{**is exempt from this policy**} unless (1) information obtained is recorded in such a matter that human subject can be identified directly or through identifiers linked to the subject **and** (2) disclosure of the human subject responses outside the research could reasonably place the subjects at risk of criminal or civil liability, or be damaging to the subjects' financial standing, employability, or reputation". Under conditions mentioned in (1) and (2), the proposal must be reviewed by an Institutional Review Board Committee.

Kindly note that the certificate does not cover permissions to access the healthcare facilities.

If we can be of further assistance, please contact us at irb@moph.gov.qa

Sincerely,

Dr. Amany Salama Dahir
Health Research Governance Department Acting Director
Health Research Governance Department
Ministry of Public Health
P.O. Box: 42

This information has been labeled as Public information
